# Pediatric Trauma Undertriage: Working Toward a Better Threshold Based on Trauma Center Resource Utilization

**DOI:** 10.3390/children13010095

**Published:** 2026-01-09

**Authors:** Caitlin J. Crosier, Amber Mehmood, Keith Thatch, David J. Cisela, Etienne E. Pracht, Christopher W. Snyder

**Affiliations:** 1 Division of Pediatric Surgery, Johns Hopkins All Children’s Hospital, St. Petersburg, FL 33701, USA; kthatch3@jhmi.edu (K.T.); csnyde21@jhmi.edu (C.W.S.); 2 College of Public Health, University of South Florida, Tampa, FL 33620, USA; amehmood@usf.edu (A.M.); epracht@usf.edu (E.E.P.); 3 Division of Trauma and Acute Care Surgery, University of Colorado, Loveland, CO 80538, USA

**Keywords:** pediatric trauma, undertriage, severity of injury, injury severity score, resource utilization

## Abstract

**Highlights:**

**What are the main findings?**

• Current trauma injury severity systems are mortality-based and are derived from adult data; therefore, they have exhibited greater accuracy for adult trauma patients compared to pediatric trauma patients.
• International Classification of Disease Critical Care Severity Score (ICASS), a resource-based metric, has greater sensitivity to predict need for pediatric trauma resources compared to the Injury Severity Score (ISS) or the International Classification of Disease (ICD) Injury Severity Score (ICISS) in injured children.

**What are the implications of the main findings?**

• Pediatric trauma systems should consider utilizing ICASS to assist in defining undertriage in pediatric trauma.
• Future studies may consider comparing ICASS to other pediatric-specific trauma severity of injury metrics to better define injury severity and trauma center resource utilization in this population.

**Abstract:**

**Background/Objectives**: Pediatric trauma systems require accurate metrics for evaluating triage decisions. Undertriage occurs when an injured child requires pediatric trauma center resources but is treated at a center lacking those resources. Current undertriage definitions utilize mortality-based scores, including the Injury Severity Score (ISS) > 15 or the International Classification of Disease (ICD) Injury Severity Score (ICISS). However, resource-based metrics like the ICD Critical Care Severity Score (ICASS) may be preferable in children. This study evaluated the relationship of ISS, ICISS and ICASS to the need for pediatric trauma resources (NFPTCR) to derive a more empiric definition of undertriage. **Methods:** The American College of Surgeons Trauma Quality Improvement Program database was queried for patients aged ≤ 15 years old. NFPTCR was defined as blood product transfusion within 4 h, invasive procedure for cardiopulmonary stabilization/contamination/bleeding within 72 h, initial admission to intensive care unit (ICU) or ICU stay ≥ 3 days, intubation, mechanical ventilation and general anesthesia ≤ 5 years old, or physical child abuse. ICASS and ICISS were derived from 2014 to 2018 datasets and applied to the 2019 dataset. The ability of ISS, ICISS and ICASS to distinguish NFPTCR patients was assessed using multivariable logistic regression and receiver–operator characteristic (ROC) analysis. **Results:** Out of 97,773 children, 15,985 (16%) were NFPTCR+. ISS, ICISS and ICASS had areas under the curve of 0.760, 0.701 and 0.812 for NFPTCR+, respectively (all *p* < 0.001). ISS had 36% sensitivity at 15; whereas ICASS had 95%, 93% and 89% sensitivity at 5, 10 and 15, respectively. **Conclusions:** ICASS was superior to ISS and ICISS for identifying NFPTCR. Consideration should be given to redefining pediatric trauma undertriage based on resource-based metrics, like ICASS.

## 1. Introduction

Trauma is a significant contributor to pediatric morbidity and mortality worldwide. Pediatric trauma system functions and standards vary significantly at prehospital, emergency department and inpatient levels. There are contributions to the patient outcome at each level, and the development of an effective, efficient system that is able to be assessed is important to progressing and optimizing care. Thus, this begins at the prehospital level. Effective prehospital field trauma triage matches injured patients’ needs to hospital capabilities. Severity of injury (SOI) is measured at three levels of care: prehospital or in the field, on hospital arrival or in the emergency department/trauma bay, and at hospital discharge to assess outcomes in relation to the overall care provided. Various schemes are employed at each level, with different metrics making scores to assess the severity of trauma and risk of need for critical care resources or mortality. At the regional trauma system level, external undertriage occurs when a patient with major trauma is taken to a center lacking the resources to meet the patient’s needs. This form of undertriage involves decisions regarding which hospital a patient should go to; it is distinct from internal undertriage, which occurs when a hospital fails to activate an appropriate level of alert or response for the patient. Overtriage occurs when a patient with minimal or low-risk injury is brought from the field (primary overtriage) or transferred (secondary overtriage) to a high-level trauma center [1]. Trauma systems generally accept higher levels of overtriage in order to avoid undertriage, since external undertriage increases risk of patient morbidity and mortality [2,3,4].

Severity of injury (SOI) metrics, such as the Injury Severity Score (ISS), are essential tools for evaluating the effectiveness of regional trauma systems. By quantifying a patient’s total burden of injury, appropriate SOI metrics provide a reference standard for retrospectively evaluating field triage schemes and care received in the hospital, accurately monitoring undertriage and comparing outcomes [5,6].

Current benchmarks of the American College of Surgeons define major trauma as an ISS greater than 15 [1]. However, this definition is derived primarily from historical adult populations and may not be optimal for injured children, and a cutoff of an ISS greater than 25 has also been proposed in pediatric trauma [7]. As a mortality-based metric, the ISS tends to underestimate the burden of injury in children, in whom the risk of mortality is far lower than that of adults [8,9]. In addition, calculation of the ISS requires trauma-specific data collection typically found only in a trauma registry, whereas most injured children receive care outside of dedicated pediatric trauma centers [10]. The pediatric traumatology field needs a sensitive, specific, simple and suitable metric that can be applied across the inclusive trauma system to all centers caring for injured children, not just high-level trauma centers that maintain a trauma registry.

The International Classification of Disease (ICD) Injury Severity Score (ICISS) uses ICD diagnosis codes to estimate the risk of mortality [11,12]. The ICD Critical Care Severity Score (ICASS) uses diagnosis codes in a similar fashion to quantify risk of utilizing critical care resources [8,9]. Both metrics have been previously validated and are easily calculated from administrative data alone [9,11,13]. ICASS has demonstrated greater ability to estimate critical care resource utilization than the ISS or ICISS are able to estimate mortality risk in pediatric trauma when compared to adult trauma patients [8,9].

Since the concept of undertriage focuses on inadequate resources to meet a patient’s needs, other authors have proposed that the definition of undertriage should be based on the patient’s risk of utilizing trauma center resources, not an arbitrary threshold risk of mortality [14,15,16,17]. Consensus-based definitions of trauma center resources have been previously published [18,19,20,21,22]. Many injured children are treated at community hospitals that do not have the resources to maintain a trauma registry and calculate ISS for each patient. Simple metrics that are predictive of trauma center resource utilization and that can be calculated based on administrative diagnosis codes alone would be valuable tools to assist with quality improvement. The purpose of this study was to compare the ability of ISS, ICISS and ICASS to distinguish the need for pediatric trauma center (PTC) resources (NFPTCR), in order to derive a better threshold for defining pediatric trauma undertriage for use in the quality improvement setting. We hypothesized that ICASS would demonstrate superior ability to identify NFPTCR compared to ISS or ICISS.

## 2. Materials and Methods

The American College of Surgeons (ACS) Trauma Quality Improvement Program (TQIP) participant user files were queried for injured patients aged ≤ 15 years old and younger treated during the years 2014 to 2019. Patients were excluded if they were aged 16 years old or older or had missing ISS. The 2014–2018 datasets were used to derive ICISS and ICASS values for each diagnosis, as described previously [8,9]. The ICISS for each ICD diagnosis code was calculated by the standard method using survival risk ratios. ICISS is reported as a decimal value, therefore the ICISS was then inverted (1-ICISS) and multiplied by 100 to create a scale so that ICISS could be compared to ISS and ICASS on the same scale. ICASS is a critical care risk ratio calculated by dividing the number of episodes of critical care services provided for an ICD diagnosis by the number of patients with each diagnosis, then multiplying this by 100. Patients are assigned the maximum ICASS value as their ICASS. For example, if a patient has two diagnoses, with ICASS risk ratios of 45 and 60, their ICASS would be 60. Scores range 0–100, with higher scores indicating greater injury severity. The ICISS survival risk ratio and ICASS critical care risk ratios derived from the 2014–2018 cohort were then applied to the 2019 patients to calculate their ICISS and ICASS based on their individual diagnosis codes. ISS for each 2019 patient was taken from the embedded TQIP variables.

Need for pediatric trauma center resources (NFPTCR) was defined as any one of the following criteria: emergent transfusion of any blood product (within 4 h of arrival): packed red blood cells, plasma, platelets, and/or cryoprecipitate; urgent need for one of the following procedures (within 72 h of arrival): tube thoracostomy, pericardiocentesis, intracranial pressure monitoring, craniotomy, laparotomy, hemorrhage control, invasive angiography, repair/resection procedure for solid organ or hollow viscus injury; any general anesthesia or mechanical ventilation in patients 5 years old or younger at any time during the index hospitalization; admission from emergency dept to intensive care unit (ICU), or any ICU stay ≥ 3 days; physical child abuse report or investigation. These criteria are listed in Table 1. These criteria were chosen based on previous studies [17,19,20]. General anesthesia in children 5 years old and younger was added based on the ACS recognition of pitfalls of anesthesia in young children and recommendations for patients 5 and younger to be cared for by a pediatric anesthesiologist [23]. Child abuse report/investigation was added to the NFPTCR criteria based on national consensus guidelines [24].

The ability of ISS, ICISS and ICASS to predict NFPTCR was assessed using univariate logistic regression modeling and receiver–operator characteristic (ROC) analysis. ROC curves were generated for ISS, ICISS and ICASS. The resulting areas under the curves (AUCs) for the ICASS and ICISS metrics were compared with ISS, which served as the reference standard. Statistical comparisons of AUCs and their 95% confidence intervals were performed using contrast estimates and chi-square tests. Sensitivity and specificity of ISS, ICISS and ICASS for predicting NFPTCR at predefined threshold values of 5, 10, 15 and 20 were calculated. Statistical analysis was carried out using SAS 9.4 (Statistical Analysis Software) (SAS Institute, Cary, NC, USA).

## 3. Results

###  Descriptive Results

There were 97,773 patients who met the inclusion criteria, of whom 15,985 (16%) utilized pediatric trauma center resources (NFPTCR+). Comparison of demographic and clinical factors for patients who utilized pediatric trauma center resources versus those who did not is shown in Table 2. All demographic and clinical variables were statistically different between NFPTCR+ and NFPTCR− groups, *p* < 0.0001, except for sex, *p* = 0.54. NFPTCR+ patients had significantly higher prevalence of penetrating, physical abuse and transport-related mechanisms. They also had a significantly higher risk of in-hospital death (5% versus 0.3%, *p* < 0.0001) and a lower likelihood of discharge home from the emergency department (1% versus 20%, *p* < 0.0001) compared to NFPTCR− patients. Among patients who did not die or were transferred to another hospital, hospital stay was significantly longer among NFPTCR+ patients (median 62.5 versus 18.0 h, *p* < 0.0001).

**Figure 1:**
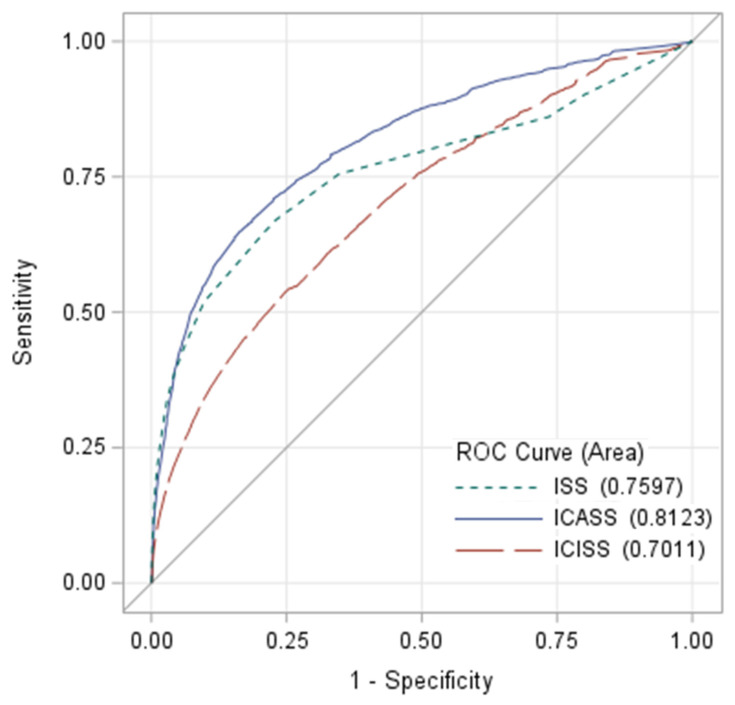
ROC curves for the ability of ISS, ICISS and ICASS to distinguish between NFPTCR+ patients vs. NFPTCR− patients.

For NFPTCR+ patients, the median ISS, ICISS and ICASS were 10, 37 and 52, respectively. Thirty-four percent of NFPTCR+ patients had ISS > 15, while 95%, 93% and 89% of NFPTCR+ patients had ICASS > 5, 10 and 15, respectively. In the ROC analysis comparing the ability of ISS, ICISS and ICASS to predict NFPTCR status, ICASS demonstrated the greatest AUC compared to ISS and ICISS (Figure 1). The AUC for ISS was 0.76 (95% confidence interval (CI) 0.755–0.765), AUC for ICISS was 0.701 (95% CI 0.696–0.706) and AUC for ICASS was 0.812 (95% CI 0.808–0.816). The difference in AUC between all three curves was statistically significant (*p* < 0.0001 for all pairwise comparisons). Table 3 provides the sensitivity and specificity of the three metrics at threshold values of 5, 10, 15 and 20. ICASS provided >95% sensitivity for detecting NFPTCR at a threshold value of 5. At its standard threshold value of 15, ISS was 36% sensitive for detecting NFPTCR.

## 4. Discussion

This study showed that when utilization of PTC resources is considered, ISS was a poor indicator of undertriage, with only 36% sensitivity at its standard threshold of 15. Put another way, about two-thirds of injured children who needed PTC resources would not have met the accepted ISS-based definition of “major trauma.” Conversely, ICASS demonstrated the highest AUC on ROC analysis and had excellent sensitivity for PTC resource utilization. ICISS, while showing similar sensitivity to ICASS at a threshold of 5, demonstrated worse specificity and an overall lower AUC on ROC comparison.

This study adds to the growing body of literature suggesting that ISS-based definitions of undertriage are inadequate, especially in pediatric patients [15,16,17]. A variety of injury severity metrics have been described for pediatric trauma, including the pediatric trauma score (PTS), trauma injury severity score (TRISS), Trauma Composite Score and rSIG (reverse Shock Index (SI) times Glasgow Coma Scale (GCS)) [6,7,25,26]. These metrics are used to assist in prehospital triage decision making, whereas the ISS, ICISS and ICASS are used to retrospectively review how well prehospital triage decisions align with in-hospital trauma care requirements and outcomes. Additionally, these metrics are focused on predicting mortality or require granular data only available in a trauma registry. Previous studies have shown that resource-based measures, such as the ICASS, may be preferable to mortality-based measures, such as the ISS or ICISS, in quantifying the impact of injury on pediatric trauma patients [8,9]. While the need for trauma intervention (NFTI) used in previous studies may be valuable for determining the level of trauma activation within a dedicated trauma center (i.e., internal triage), it is expected to lack sufficient sensitivity to be used as a benchmark for determining appropriate triage at the regional trauma system level (i.e., external primary triage) [17].

Like ISS, ICASS is calculated after the patient’s injuries are known and is not intended to serve as a field triage tool in and of itself. However, defining undertriage using a pediatric-appropriate, resource utilization-based metric such as ICASS is necessary to advance comparative research of pediatric field triage protocols [5]. Without an appropriate “back-end” definition of undertriage, field triage protocols have no standard against which to compare themselves. This study shows that ICASS is superior to ISS in defining thresholds for pediatric undertriage and paves the way for necessary future studies. The criteria used to calculate ICASS are easily accessible metrics that can be calculated by a simple query of hospital electronic medical records or billing data.

At a threshold value of 5, ICASS demonstrates 24% specificity, corresponding to a high overtriage rate. Many non-modifiable factors contribute to overtriage. External primary overtriage is likely inevitable, since the PTC typically has a dual role as both a pediatric community hospital as well as a trauma center. External secondary overtriage, in which a low-risk patient is transferred to the PTC from another center, is a multifactorial problem known to occur at high rates [27,28]. The optimal ICASS threshold depends on the primary goal of its application. If the goal is to keep undertriage rates below 5%, an ICASS of 5 would be appropriate. If a closer balance between overtriage and undertriage is desired, a higher ICASS threshold could be selected as shown in Table 3. Selecting a higher ICASS threshold, such as 15, would maintain a sensitivity of 89%, providing reasonable screening or triage ability, and decrease the rate of overtriage substantially, at a specificity of 46%. Better refining triage criteria may have positive financial implications for healthcare systems, insurance companies and patients since appropriately triaging patients will be the most effective and efficient way to provide the correct level and location of care. Additional studies may be needed to refine ICASS thresholds for primary vs. secondary and external vs. internal triage.

This study must be interpreted in the context of some limitations. We did not derive a truly empirical new system or model for defining undertriage; rather, we empirically explored different numeric thresholds within three existing SOI scales. The NFPTCR definition used in this study, while based on a synthesis of the previous literature, is not standardized and has not undergone broad consensus validation. Since the NFPTCR standard for undertriage between centers is resource-based, using the resource-based ICASS to define undertriage may raise concerns for circular reasoning—i.e., need for resources predicting need for resources. However, ICASS estimates the overall risk of requiring any critical care services, while NFPTCR is based on trauma-related resources. Only some of the NFPTCR criteria are explicitly associated with aspects of critical care being ICU admission or ICU stay, while all other criteria are utilized in cases that exist along a spectrum of severity, and not all patients requiring these interventions will necessarily require critical care resources beyond the initial trauma resuscitation or operating room. While there is some expected overlap, ICASS and NFPTCR are ultimately not measuring the same thing. In addition, variables that predict mortality and resource utilization typically exhibit strong collinearity, so this circularity concern would apply to any candidate SOI metric. These scoring systems were developed from a large dataset of trauma centers in North America but may not be broadly applicable to other countries and practice settings. Finally, ICD code-based metrics are subject to coding inaccuracies and hospital-level variability.

Future studies should compare the ICASS to other pediatric SOI metrics. Consensus is also needed in the pediatric trauma community to define the unique scope and contribution of resources specific to the pediatric trauma center.

## 5. Conclusions

The current ISS-based definition of undertriage is inadequate for injured children and should be replaced with a metric empirically demonstrated to be more applicable to the pediatric population. Undertriage metrics used to compare different centers (i.e., at the trauma system level) should emphasize the multidisciplinary resources of the center as a whole. ICASS is simple, sensitive and calculable for any center based on administrative data alone. ICASS achieves 95% sensitivity at a suggested threshold of 5, and can be considered as a candidate benchmark for assessing trauma system-level undertriage in the retrospective quality improvement setting.

## Figures and Tables

**Table 1:** Criteria used to define NFPTCR+.

Criterion Name	Criterion Description	Timeframe from Arrival
Transfusion	Emergent transfusion of any blood product: packed red blood cells, plasma, platelets, and/or cryoprecipitate	4 h
Urgent procedure	Procedural intervention: tube thoracostomy, pericardiocentesis, intracranial pressure monitoring, craniotomy, laparotomy, hemorrhage control, invasive angiography, repair/resection procedure for solid organ or hollow viscus injury	72 h
Anesthesia ≤ 5 yrs	Any general anesthesia or mechanical ventilation in patients 5 years old or younger	Any
Intensive care unit	Admission from emergency dept to ICU, or any ICU stay ≥ 3 days	Any
Child abuse	Physical child abuse report or investigation	Any

NFPTCR, need for pediatric trauma center resources; ICU, intensive care unit.

**Table 2:** Demographic and clinical data of NFPTCR+ and NFPTCR− groups.

Variable	NFPTCR+ (n = 15,985)	NFPTCR− (n = 81,788)	*p*-Value
Age, years	7 (3–13)	8 (4–12)	<0.0001
Sex, female	10,145 (63)	51,688 (63)	0.54
*Injury mechanism*			<0.0001
Abuse/neglect	1166 (7)	119 (0.2)	
Gunshot/stab wound	766 (5)	1118 (1)	
Transport-related	6671 (42)	24,214 (30)	
Other blunt accidental/undetermined	7382 (46)	56,337 (69)	
Received as interfacility transfer	6658 (42)	31,247 (38)	<0.0001
Arrived in cardiac arrest, or history of prehospital arrest	632 (4)	354 (0.4)	<0.0001
*Treating center pediatric verification level*			<0.0001
Level 1	5194 (32)	31,124 (38)	
Level 2	2623 (16)	10,991 (13)	
Non-verified/designated	8168 (51)	39,673 (49)	
*Outcomes*			
Discharge home from emergency department	216 (1)	16,079 (20)	<0.0001
Transfer to another hospital	927 (6)	9696 (12)	<0.0001
Death	850 (5)	214 (0.3)	<0.0001
Intensive care unit days among pts not transferred/deceased	2 (0–3)	0 (0–0)	<0.0001
Hospital length of stay among pts not transferred/deceased, hours	62.5 (28.3–139.1)	18.0 (7.0–33.3)	<0.0001
*Metrics*			
Injury Severity Score (ISS)	10 (5–17)	4 (2–5)	<0.0001
ICD (ICD) Injury Severity Score (ICISS)	37 (19–59)	18 (8–34)	<0.0001
ICD Critical Care Severity Score (ICASS)	52 (28–67)	17 (5–29)	<0.0001
ISS > 15	5447 (34)	2413 (3)	
ICISS > 10	13,971 (87)	56,843 (70)	
ICASS > 5	15,241 (95)	62,567 (77)	
ICASS > 10	14,848 (93)	52,835 (65)	
ICASS > 15	14,161 (89)	44,355 (54)	

Values given as n (%) for categorical and median (interquartile range) for continuous variables, respectively. Statistically significant differences between groups (*p* < 0.0001) for all variables except sex (*p* = 0.54). Groups compared using chi-square tests for categorical variables; Kruskal–Wallis tests for continuous variables. NFPTCR, need for pediatric trauma center resources; ISS, injury severity score; ICISS, International Classification of Disease injury severity score; ICASS, International Classification of Disease critical care severity score.

**Table 3:** Sensitivity, specificity and receiver–operator characteristic curves for thresholds of ISS, ICISS and ICASS to predict utilization of pediatric trauma center resources.

Threshold Value	5	10	15	20		
	Sensitivity/Specificity	Sensitivity/ Specificity	Sensitivity/ Specificity	Sensitivity/ Specificity	AUC (95% CI)	*p* *
ISS	75.5/65.4	51.9/90.3	36.3/96.5	22.6/98.8	0.760 (0.755–0.765)	Ref
ICISS	96.4/15.7	87.4/30.5	80.2/42.5	74.0/52.5	0.701 (0.696–0.706)	<0.0001
ICASS	95.3/23.6	92.9/35.3	88.7/45.6	85.4/54.9	0.812 (0.808–0.816)	<0.0001

ISS, Injury Severity Score; ICISS, International Classification of Disease injury severity score; ICASS, International Classification of Disease critical care severity score; AUC, area under the receiver–operator characteristic curve; CI, confidence interval; Ref, reference. * *p*-value for comparison of AUCs.

## Data Availability

The TQIP participant user files (PUF) utilized for this study were provided from the TQIP database upon formal request to be used for directed research purposes and approved and provided for the purpose of this study only by the American College of Surgeons Committee on Trauma (ACS COT). As such, the data files are not to be shared. Requests for further information regarding data sharing should be directed to the corresponding author.

## Abbreviations

The following abbreviations are used in this manuscript:

ACS	American College of Surgeons
AUC	area under the curve
ICASS	International Classification of Disease Critical Care Severity Score
ICD	International Classification of Disease
ICISS	International Classification of Disease injury severity score
ISS	injury severity score
NFPTCR	need for pediatric trauma resources
NFTI	need for trauma intervention
PTC	pediatric trauma center
PTS	pediatric trauma score
ROC	receiver–operator characteristic
rSIG	reverse Shock Index (SI) times Glasgow Coma Scale (GCS)
SOI	severity of injury
TQIP	Trauma Quality Improvement Program
TRISS	trauma injury severity score
